# Supplementary material for: The Small RNA Universe of Capitella teleta
Source: Front Mol Biosci. 2022 Feb 25;9:802814. doi: 10.3389/fmolb.2022.802814 (PMC8915122; doi:10.3389/fmolb.2022.802814)
Supplement: Supplementary file 1 [file DataSheet1.ZIP › Supplement/known/CAPTEscaffold_558_23955.pdf]

Provisional ID	:	CAPTEscaffold_558_23955
Score total	:	55543.8
Score for star read(s)	:	3.9
Score for read counts	:	55533.3
Score for mfe	:	2
Score for randfold	:	1.6
Score for cons. seed	:	3
Total read count	:	108938
Mature read count	:	93805
Loop read count	:	1
Star read count	:	15132

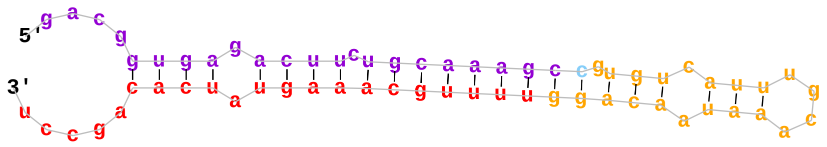

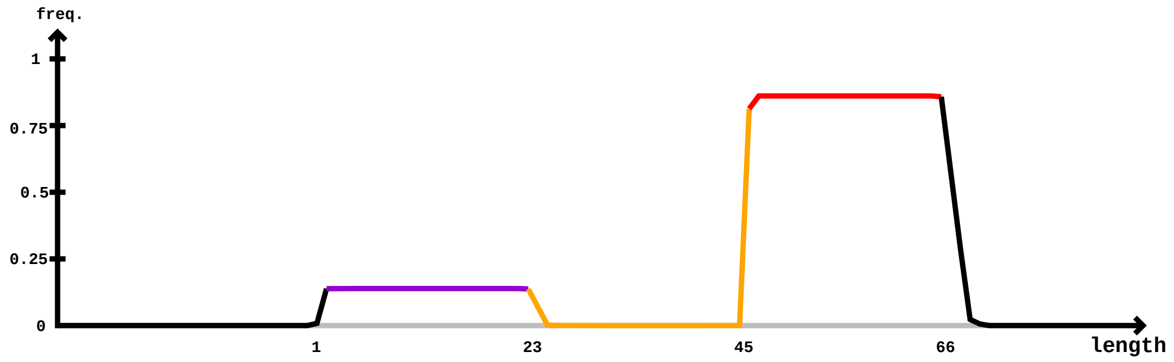

Star **Mature**

```
5'- cagcgcugcugcugcuacugcaggccgacggugagacuucugcaaagccgugucauuugcaaauaacagguuuugcaaaguaucacagccugucugcgguauggcacaccg  -3'   obs
    cagcgcugcugcugcuacugcaggccgacggugagacuucugcaaagccgugucauuugcaaauaacagguuuugcaaaguaucacagccugucugcgguauggcacaccg         exp
    cagcgcugcugcugcuacugcaggccgacggugagacuucugcaaagccgugucauuugcaaauaacagguuuugcaaaguaucacagccugucugcgguauggcacaccg         known
    ...((.((.(((((.(((((((((.(.(.((((.((((.((((((((.(((.(((....))).))))))))))))))).)))).).).))))))))))))))).))   reads   mm   sample

    .....cugcugcugcuacugcaggcc.....................................................................................   2   0   seq
    .......gcugcugcuacugcaggcc.....................................................................................   3   0   seq
    .........................cgacggugagacuucugca...................................................................   1   0   seq
    .........................cgacggugagacuucugcaa..................................................................   2   0   seq
    .........................cgacggugagacuucugcaaag................................................................   10   0   seq
    .........................Ngacggugagacuucugcaaagc...............................................................   2   1   seq
    .........................cgacggugagacuucugcaaagA...............................................................   3   1   seq
    .........................cgacggugagacuucugcaaagc...............................................................   867   0   seq
    .........................Ugacggugagacuucugcaaagc...............................................................   1   1   seq
    .........................cgaUggugagacuucugcaaagc...............................................................   1   1   seq
    .........................cgacggAgagacuucugcaaagc...............................................................   1   1   seq
    .........................cgacggugagacuucugcaaagcc..............................................................   1   0   seq
    ..........................gacggugagacuucugcaaa.................................................................   18   0   seq
    ..........................gacggugagacAucugcaaag................................................................   1   1   seq
    ..........................gGcggugagacuucugcaaag................................................................   2   1   seq
    ..........................gacggugagacuucugcaaag................................................................   157   0   seq
    ..........................gacggugagacuucugcGaagc...............................................................   2   1   seq
    ..........................gacggAgagacuucugcaaagc...............................................................   7   1   seq
    ..........................gacggugagacuucugcaaagU...............................................................   1   1   seq
    ..........................gacggugagUcuucugcaaagc...............................................................   1   1   seq
    ..........................gacggugagacuucugcaaaUc...............................................................   2   1   seq
    ..........................Aacggugagacuucugcaaagc...............................................................   12   1   seq
    ..........................gacAgugagacuucugcaaagc...............................................................   6   1   seq
    ..........................Uacggugagacuucugcaaagc...............................................................   12   1   seq
    ..........................gacggugagacGucugcaaagc...............................................................   1   1   seq
    ..........................gacggugUgacuucugcaaagc...............................................................   1   1   seq
    ..........................Cacggugagacuucugcaaagc...............................................................   4   1   seq
    ..........................gacggugGgacuucugcaaagc...............................................................   1   1   seq
    ..........................gacggugagacuucAgcaaagc...............................................................   6   1   seq
    ..........................gacggugagacuucugcaGagc...............................................................   2   1   seq
    ..........................gacggugagacuucuAcaaagc...............................................................   1   1   seq
```

```
                         Star                                      Mature
cagcgcugcugcugcuacugcaggccgacggugagacuucugcaaagccgugucauuugcaaauaacagguuuugcaaaguaucacagccugucugcgguauggcacaccg
..........................gacggugagacCucugcaaagc...............................................................  1     1     seq
..........................gacggugagacuAcugcaaagc...............................................................  1     1     seq
..........................gacggugagacuucugcaUagc...............................................................  1     1     seq
..........................gacggugagaAuucugcaaagc...............................................................  1     1     seq
..........................gacgguAagacuucugcaaagc...............................................................  4     1     seq
..........................gacgUugagacuucugcaaagc...............................................................  1     1     seq
..........................gacggugagaGuucugcaaagc...............................................................  1     1     seq
..........................gGcggugagacuucugcaaagc...............................................................  83    1     seq
..........................gacggugaAacuucugcaaagc...............................................................  1     1     seq
..........................gacggugagacuucugcaaagA...............................................................  29    1     seq
..........................gacggugagacuucugcaaGgc...............................................................  1     1     seq
..........................gacggugagacuucugcaaagc...............................................................  9426  0     seq
..........................gacCgugagacuucugcaaagc...............................................................  1     1     seq
..........................gacggugagacuucugGaaagc...............................................................  1     1     seq
..........................gacggugagacuucuCcaaagc...............................................................  4     1     seq
..........................gacggugagacuucugAaaagc...............................................................  4     1     seq
..........................gacggugagGcuucugcaaagc...............................................................  2     1     seq
..........................gacgAugagacuucugcaaagc...............................................................  3     1     seq
..........................Nacggugagacuucugcaaagc...............................................................  1     1     seq
..........................gacggugagacuucGgcaaagc...............................................................  1     1     seq
..........................gaAggugagacuucugcaaagc...............................................................  4     1     seq
..........................gacggugagacAucugcaaagc...............................................................  1     1     seq
..........................gacggugagacuucCgcaaagc...............................................................  1     1     seq
..........................gacUgugagacuucugcaaagc...............................................................  1     1     seq
..........................gacggugagacuuUugcaaagcc..............................................................  1     1     seq
..........................gacgAugagacuucugcaaagcc..............................................................  2     1     seq
..........................gacggugagacuucugcaaagUc..............................................................  1     1     seq
..........................Aacggugagacuucugcaaagcc..............................................................  5     1     seq
..........................gacggugagacuucugcaaagcA..............................................................  3     1     seq
..........................gacgguCagacuucugcaaagcc..............................................................  1     1     seq
..........................gacggugagacuAcugcaaagcc..............................................................  2     1     seq
..........................gacggugagacuucugcaaGgcc..............................................................  1     1     seq
..........................Uacggugagacuucugcaaagcc..............................................................  12    1     seq
..........................gacAgugagacuucugcaaagcc..............................................................  5     1     seq
..........................gGcggugagacuucugcaaagcc..............................................................  39    1     seq
..........................gacggugagacGucugcaaagcc..............................................................  1     1     seq
..........................gacggugagacuucAgcaaagcc..............................................................  2     1     seq
..........................gacggugagaUuucugcaaagcc..............................................................  1     1     seq
..........................Cacggugagacuucugcaaagcc..............................................................  3     1     seq
..........................gacggugagacuucugcaaagcc..............................................................  4089  0     seq
..........................gacgCugagacuucugcaaagcc..............................................................  1     1     seq
..........................gacggAgagacuucugcaaagcc..............................................................  6     1     seq
..........................gacggugagacuucugcGaagcc..............................................................  3     1     seq
..........................gacgguAagacuucugcaaagcc..............................................................  6     1     seq
..........................gacggCgagacuucugcaaagcc..............................................................  1     1     seq
..........................gacggugagacuucugcCaagcc..............................................................  1     1     seq
..........................gacggugagacuucugcaaagcU..............................................................  1     1     seq
..........................gacggugagaAuucugcaaagcc..............................................................  1     1     seq
..........................gacggugagacuucugcaaagAc..............................................................  2     1     seq
..........................gacggugagacuucugcaaagccA.............................................................  4     1     seq
..........................gacggugagacuucugcaaagccg.............................................................  3     0     seq
..........................gacggugagacuucugcaaagccU.............................................................  159   1     seq
...........................acggugagacuucugcaaagc...............................................................  6     0     seq
...........................acggugagacuucugcaaagcc..............................................................  38    0     seq
...........................acggugagacuucugcaaagccU.............................................................  1     1     seq
.............................ggugagacuucugcaaagccgug...........................................................  3     0     seq
.............................ggugagacuucugcaaagccgugu..........................................................  21    0     seq
.............................ggugagacuucugcaaagccguguU.........................................................  2     1     seq
.............................ggugagacuucugcaaagccguguc.........................................................  1     0     seq
.............................ggugagacuucugcaaagccgugucUu.......................................................  1     1     seq
..............................gugagacuucugcaaagccgug...........................................................  2     0     seq
...............................ugagacuucugcaaagccgugu..........................................................  2     0     seq
...............................ugagacuucugcaaagccgugucU........................................................  2     1     seq
...............................ugagacuucugcaaagccguguca........................................................  2     0     seq
.................................................gugucauuugcaaauaacagg.........................................  1     0     seq
..................................................................cagguuuugcaaaguaucacagc......................  6     0     seq
...................................................................agguuuugcaaaguaucacagc......................  2     0     seq
......................................................................uuuugcaaaguaucacagc......................  4     0     seq
......................................................................uuuugAaaaguaucacagcc.....................  1     1     seq
......................................................................uuuugcaaaguaucGcagcc.....................  1     1     seq
```

Star Mature

```
cagcgcugcugcugcuacugcaggccgacggugagacuucugcaaagccgugucauuugcaaauaacagguuuugcaaaguaucacagccugucugcgguauggcacaccg
......................................................................uuuugcaaaguaucaUagcc.....................	1	1	seq
......................................................................uuuugcaaaguaucacagcc.....................	302	0	seq
......................................................................uuuugcaaGguaucacagcc.....................	1	1	seq
......................................................................uuAugcaaaguaucacagcc.....................	1	1	seq
......................................................................uuuugcaaaguGucacagcc.....................	1	1	seq
......................................................................uuuugcaaaguaAcacagcc.....................	1	1	seq
......................................................................Nuuugcaaaguaucacagcc.....................	1	1	seq
......................................................................uuuugcaaaguaucacaAccu....................	3	1	seq
......................................................................uuuuCcaaaguaucacagccu....................	3	1	seq
......................................................................uuuugcGaaguaucacagccu....................	11	1	seq
......................................................................uuuCgcaaaguaucacagccu....................	2	1	seq
......................................................................uuuugcaaaguGucacagccu....................	5	1	seq
......................................................................uuuugcaaaguauUacagccu....................	22	1	seq
......................................................................uuuugcaaaguaucUcagccu....................	7	1	seq
......................................................................uuuugcaaaguaucacagccG....................	204	1	seq
......................................................................uuuugcaaaguaucacagUcu....................	2	1	seq
......................................................................uuuugcaaaguaucacagccA....................	97	1	seq
......................................................................uuuugcaaaAuaucacagccu....................	1	1	seq
......................................................................uuuugcaaGguaucacagccu....................	55	1	seq
......................................................................uuuugAaaaguaucacagccu....................	9	1	seq
......................................................................uuuuAcaaaguaucacagccu....................	3	1	seq
......................................................................uuuugcaaaguaucacagccC....................	8	1	seq
......................................................................uuuAgcaaaguaucacagccu....................	10	1	seq
......................................................................uuuugcaaaguUucacagccu....................	4	1	seq
......................................................................uuuugcaaaguaucacagcUu....................	6	1	seq
......................................................................uuuugcaUaguaucacagccu....................	5	1	seq
......................................................................uuAugcaaaguaucacagccu....................	26	1	seq
......................................................................uuuugcaaUguaucacagccu....................	1	1	seq
......................................................................Nuuugcaaaguaucacagccu....................	10	1	seq
......................................................................uuuugcaaaguaucacUgccu....................	2	1	seq
......................................................................uuuugcCaaguaucacagccu....................	1	1	seq
......................................................................uuuugcaaaguaGcacagccu....................	3	1	seq
......................................................................uuuugcaaaguaucacagcGu....................	3	1	seq
......................................................................uuuugcaaaguaucacagccu....................	32627	0	seq
......................................................................uuuGgcaaaguaucacagccu....................	3	1	seq
......................................................................uuuugcaaaguaucaGagccu....................	2	1	seq
......................................................................uuuugcaaaguaucacagGcu....................	3	1	seq
......................................................................uuuugcaaagAaucacagccu....................	7	1	seq
......................................................................uuuugcaaagGaucacagccu....................	1	1	seq
......................................................................uuuugUaaaguaucacagccu....................	5	1	seq
......................................................................Cuuugcaaaguaucacagccu....................	23	1	seq
......................................................................uuuugcaaaguaucacaUccu....................	3	1	seq
......................................................................uuuugcaaaguaucaAagccu....................	5	1	seq
......................................................................uuuugcaaaguauAacagccu....................	7	1	seq
......................................................................uuuugGaaaguaucacagccu....................	2	1	seq
......................................................................uNuugcaaaguaucacagccu....................	3	1	seq
......................................................................uuuugcaaaguaCcacagccu....................	8	1	seq
......................................................................uuuugcaaaguaucacagcAu....................	13	1	seq
......................................................................uuuuUcaaaguaucacagccu....................	4	1	seq
......................................................................uuuugcaGaguaucacagccu....................	17	1	seq
......................................................................Guuugcaaaguaucacagccu....................	22	1	seq
......................................................................uuuugcaaaguaAcacagccu....................	6	1	seq
......................................................................uuuugcaaaguaucacaCccu....................	3	1	seq
......................................................................uuuugcaaagCaucacagccu....................	5	1	seq
......................................................................uuuugcaaaguaucGcagccu....................	5	1	seq
......................................................................uuuugcaaaguaucCcagccu....................	2	1	seq
......................................................................uuuugcaaaguaucacagAcu....................	7	1	seq
......................................................................uuCugcaaaguaucacagccu....................	1	1	seq
......................................................................uuuugcaaaguaucacGgccu....................	7	1	seq
......................................................................uCuugcaaaguaucacagccu....................	8	1	seq
......................................................................uAuugcaaaguaucacagccu....................	19	1	seq
......................................................................Auuugcaaaguaucacagccu....................	122	1	seq
......................................................................uuuugcaaaguaucaUagccu....................	4	1	seq
......................................................................uuCugcaaaguaucacagccug...................	2	1	seq
......................................................................uuuugcaaUguaucacagccug...................	4	1	seq
......................................................................uuuugcaaaguaucaUagccug...................	3	1	seq
......................................................................uuuugUaaaguaucacagccug...................	6	1	seq
......................................................................uNuugcaaaguaucacagccug...................	3	1	seq
......................................................................uuuugcaaaguaGcacagccug...................	2	1	seq
......................................................................uuuugcaaagGaucacagccug...................	2	1	seq
```

```
                          Star                                          Mature
cagcgcugcugcugcuacugcaggccgacggugagacuucugcaaagccgugucauuugcaaauaacagguuuugcaaaguaucacagccugucugcgguauggcacaccg
......................................................................uuuugcaaagAaucacagccug...................   7       1   seq
......................................................................Nuuugcaaaguaucacagccug...................   15      1   seq
......................................................................uuuugcaaaguaucaGagccug...................   1       1   seq
......................................................................uuAugcaaaguaucacagccug...................   17      1   seq
......................................................................uAuugcaaaguaucacagccug...................   14      1   seq
......................................................................uuuugcGaaguaucacagccug...................   8       1   seq
......................................................................uuuugcaaaguaucacagccAg...................   7       1   seq
......................................................................uuuugcaaaUuaucacagccug...................   3       1   seq
......................................................................uuuugcaaaguaucacaAccug...................   6       1   seq
......................................................................uuuugcaaaguaucUcagccug...................   2       1   seq
......................................................................uuuugcaaaguaucacagGcug...................   1       1   seq
......................................................................uuuugcaaaguaucacaUccug...................   1       1   seq
......................................................................uuuugcaUaguaucacagccug...................   4       1   seq
......................................................................uuuugcaaagCaucacagccug...................   3       1   seq
......................................................................uuuugcaaaguaucacGgccug...................   3       1   seq
......................................................................uuuugcaGaguaucacagccug...................   12      1   seq
......................................................................uuuugcaaaCuaucacagccug...................   1       1   seq
......................................................................uuuugcaaaguaucacagccGg...................   198     1   seq
......................................................................uuuAgcaaaguaucacagccug...................   15      1   seq
......................................................................uuuugGaaaguaucacagccug...................   2       1   seq
......................................................................uuuugcaaaguaucacagccCg...................   3       1   seq
......................................................................uuuCgcaaaguaucacagccug...................   5       1   seq
......................................................................uuuugcaaaguauUacagccug...................   13      1   seq
......................................................................uuuugcaaaguaucacagccuU...................   387     1   seq
......................................................................uuuugcaaaguaucacaCccug...................   2       1   seq
......................................................................uuuugcaaaguaucaAagccug...................   4       1   seq
......................................................................uuuuUcaaaguaucacagccug...................   3       1   seq
......................................................................uuuugcaaaguaucacagcAug...................   11      1   seq
......................................................................Cuuugcaaaguaucacagccug...................   35      1   seq
......................................................................uuuugcaaaguaucacagUcug...................   1       1   seq
......................................................................uuuGgcaaaguaucacagccug...................   1       1   seq
......................................................................uuuugAaaaguaucacagccug...................   7       1   seq
......................................................................uuuuAcaaaguaucacagccug...................   4       1   seq
......................................................................uuuuCcaaaguaucacagccug...................   1       1   seq
......................................................................uuuugcaaaguaucacagcGug...................   6       1   seq
......................................................................uuuugcaaaguaucacagccug...................   27120   0   seq
......................................................................uuuugcaaaguaucGcagccug...................   5       1   seq
......................................................................uuuugcaaaAuaucacagccug...................   3       1   seq
......................................................................uuuugcaaaguGucacagccug...................   5       1   seq
......................................................................uuuugcaaaguaucacagccuA...................   13      1   seq
......................................................................Auuugcaaaguaucacagccug...................   91      1   seq
......................................................................uuuugcUaaguaucacagccug...................   2       1   seq
......................................................................uuuugcaaaguaAcacagccug...................   3       1   seq
......................................................................Guuugcaaaguaucacagccug...................   21      1   seq
......................................................................uCuugcaaaguaucacagccug...................   3       1   seq
......................................................................uuuugcaaaguaucacUgccug...................   1       1   seq
......................................................................uuuugcaaaguaucacagccuC...................   2       1   seq
......................................................................uGuugcaaaguaucacagccug...................   1       1   seq
......................................................................uuuugcaaaguaucacagAcug...................   5       1   seq
......................................................................uuuugcaaaguaucacagcUug...................   4       1   seq
......................................................................uuuugcaaGguaucacagccug...................   119     1   seq
......................................................................uuuugcaaaguauAacagccug...................   3       1   seq
......................................................................uuuugcaaaguaucacagGcugu..................   2       1   seq
......................................................................uuuugcaaaguGucacagccugu..................   6       1   seq
......................................................................uuuugcaaaUuaucacagccugu..................   1       1   seq
......................................................................uuuugcaaaguaucacagccCgu..................   3       1   seq
......................................................................Guuugcaaaguaucacagccugu..................   15      1   seq
......................................................................uNuugcaaaguaucacagccugu..................   2       1   seq
......................................................................uuuugcaaaguaucCcagccugu..................   1       1   seq
......................................................................uuuugcaaaguauUacagccugu..................   5       1   seq
......................................................................uuuugcaaaguaCcacagccugu..................   3       1   seq
......................................................................Nuuugcaaaguaucacagccugu..................   4       1   seq
......................................................................uuuugcaaaguauGacagccugu..................   2       1   seq
......................................................................uCuugcaaaguaucacagccugu..................   2       1   seq
......................................................................uuuugcaaagGaucacagccugu..................   1       1   seq
......................................................................uuuugcaaaguaAcacagccugu..................   4       1   seq
......................................................................Auuugcaaaguaucacagccugu..................   83      1   seq
......................................................................uuuugcaaaguaucacagccuCu..................   4       1   seq
......................................................................uuuugcaaaguaGcacagccugu..................   1       1   seq
......................................................................uuuugcUaaguaucacagccugu..................   5       1   seq
```

Star Mature

```
cagcgcugcugcugcuacugcaggccgacggugagacuucugcaaagccgugucauuugcaaauaacagguuuugcaaaguaucacagccugucugcgguauggcacaccg
......................................................................uuuuUcaaaguaucacagccugu..................   3      1   seq
......................................................................uuuCgcaaaguaucacagccugu..................   5      1   seq
......................................................................uuuugcaaaCuaucacagccugu..................   1      1   seq
......................................................................uuuugcaaaguaucacagcUugu..................   3      1   seq
......................................................................uuuugGaaaguaucacagccugu..................   2      1   seq
......................................................................uuuugcaaaguaucacagccGgu..................   3      1   seq
......................................................................uuuugcaaagAaucacagccugu..................   9      1   seq
......................................................................uuuAgcaaaguaucacagccugu..................   16     1   seq
......................................................................uuuugcaaaguaucacaAccugu..................   1      1   seq
......................................................................uuuugcaaaguaucacagccugu..................   23454  0   seq
......................................................................uuAugcaaaguaucacagccugu..................   8      1   seq
......................................................................uuCugcaaaguaucacagccugu..................   2      1   seq
......................................................................Cuuugcaaaguaucacagccugu..................   24     1   seq
......................................................................uuuugcaaaguaucacGgccugu..................   4      1   seq
......................................................................uuuuCcaaaguaucacagccugu..................   5      1   seq
......................................................................uuuugcaaUguaucacagccugu..................   1      1   seq
......................................................................uuuugcaaaguaucacagccugA..................   291    1   seq
......................................................................uuuugcaaagCaucacagccugu..................   3      1   seq
......................................................................uuuugcaaaguauAacagccugu..................   2      1   seq
......................................................................uuuugcaaaguaucGcagccugu..................   6      1   seq
......................................................................uuuugcaaaguaucaUagccugu..................   3      1   seq
......................................................................uuuugAaaaguaucacagccugu..................   7      1   seq
......................................................................uuuugcaaaguaucacaUccugu..................   2      1   seq
......................................................................uuuugcaaaguaucacUgccugu..................   2      1   seq
......................................................................uuuugcaaGguaucacagccugu..................   45     1   seq
......................................................................uuuugcaaaAuaucacagccugu..................   3      1   seq
......................................................................uuuugcaaaguaucaAagccugu..................   1      1   seq
......................................................................uuuugcaGaguaucacagccugu..................   16     1   seq
......................................................................uAuugcaaaguaucacagccugu..................   16     1   seq
......................................................................uuuugcaaaguUucacagccugu..................   1      1   seq
......................................................................uuuugcGaaguaucacagccugu..................   6      1   seq
......................................................................uuuugcaaaguaucacagccugG..................   100    1   seq
......................................................................uuuugcaaaguaucacagAcugu..................   6      1   seq
......................................................................uuuugcaaaguaucacagcAugu..................   10     1   seq
......................................................................uuuAcaaaguaucacagccugu..................   3      1   seq
......................................................................uuuugcaaaguaucacagUcugu..................   2      1   seq
......................................................................uuuugcaaaguaucacagccugC..................   9      1   seq
......................................................................uuuugcaaaguaucacagccuAu..................   3      1   seq
......................................................................uuuugcaaaguaucacagccuUu..................   51     1   seq
......................................................................uuuugcaaaguaucUcagccugu..................   2      1   seq
......................................................................uuuGgcaaaguaucacagccugu..................   3      1   seq
......................................................................uuuugcaaaguaucacagccAgu..................   9      1   seq
......................................................................uuuugcaaaguaucacagcGugu..................   1      1   seq
......................................................................uuuugcaaaguaucacagccuguA.................   75     1   seq
......................................................................uuuugcaaaguaucacagccuguU.................   1620   1   seq
......................................................................uuuugcaaaguaucacagccuguc.................   2      0   seq
......................................................................uuuugcaaaguaucacagccugucu................   3      0   seq
......................................................................uuuugcaaaguaucacagccuguUu................   502    1   seq
......................................................................uuuugcaaaguaucacagccuguAu................   80     1   seq
.......................................................................uuugcaaaguaucacagcc.....................   2      0   seq
.......................................................................uuugcaaGguaucacagcc.....................   1      1   seq
.......................................................................uuugcaaaguaucacagccu....................   240    0   seq
.......................................................................uuugcaaGguaucacagccu....................   5      1   seq
.......................................................................Nuugcaaaguaucacagccu....................   1      1   seq
.......................................................................uuugAaaaguaucacagccu....................   1      1   seq
.......................................................................Auugcaaaguaucacagccu....................   2      1   seq
.......................................................................uuCgcaaaguaucacagccug...................   1      1   seq
.......................................................................uuugcaaGguaucacagccug...................   5      1   seq
.......................................................................Guugcaaaguaucacagccug...................   1      1   seq
.......................................................................uuugcaaaguaucacagccGg...................   3      1   seq
.......................................................................uuugcaaaguaucacagccug...................   384    0   seq
.......................................................................uuugcaaaguaucacagccuU...................   12     1   seq
.......................................................................uAugcaaaguaucacagccug...................   1      1   seq
.......................................................................uuugcaaaguaucacagcUugu..................   1      1   seq
.......................................................................uuugcaaGguaucacagccugu..................   33     1   seq
.......................................................................Cuugcaaaguaucacagccugu..................   1      1   seq
.......................................................................uuugcaaaguaucacagccugu..................   4247   0   seq
.......................................................................uAugcaaaguaucacagccugu..................   2      1   seq
.......................................................................uuugcaaaguauAacagccugu..................   1      1   seq
.......................................................................uuugcaaaguaucUcagccugu..................   1      1   seq
```

```
                          Star                                            Mature
cagcgcugcugcugcuacugcaggccgacggugagacuucugcaaagccgugucauuugcaaauaacagguuugcaaaguaucacagccugucugcgguauggcacaccg
......................................................................uuugcaaaguaucacagccugA..................   25     1     seq
......................................................................uuAgcaaaguaucacagccugu..................   4      1     seq
......................................................................uuugcaaaguaucacagccugG..................   20     1     seq
......................................................................uuGgcaaaguaucacagccugu..................   1      1     seq
......................................................................uuugcaGaguaucacagccugu..................   3      1     seq
......................................................................Auugcaaaguaucacagccugu..................   11     1     seq
......................................................................uuugcGaaguaucacagccugu..................   1      1     seq
......................................................................uuugcaaaguauUacagccugu..................   1      1     seq
......................................................................uuugcaaaguaucacGgccugu..................   2      1     seq
......................................................................uCugcaaaguaucacagccugu..................   1      1     seq
......................................................................uuuCcaaaguaucacagccugu..................   1      1     seq
......................................................................uuugcaaaguaAcacagccugu..................   1      1     seq
......................................................................uuugcaaUguaucacagccugu..................   1      1     seq
......................................................................uuugcaaaguaucacagccCgu..................   1      1     seq
......................................................................uuugcaaaguaucacUgccugu..................   1      1     seq
......................................................................uuugcaaaguaucacagccugC..................   1      1     seq
......................................................................uuugcaaaguaucacagccGgu..................   1      1     seq
......................................................................uuugcaaagAaucacagccugu..................   3      1     seq
......................................................................uuugUaaaguaucacagccugu..................   1      1     seq
......................................................................Guugcaaaguaucacagccugu..................   1      1     seq
......................................................................Nuugcaaaguaucacagccugu..................   1      1     seq
......................................................................uuugcaaaguUucacagccugu..................   1      1     seq
......................................................................uuugcaaaguaucacagGcugu..................   1      1     seq
......................................................................uuugcaaaguaucacagccuguA.................   8      1     seq
......................................................................uuugcaaaguaucacagccuguU.................   141    1     seq
......................................................................uuugcaaaguaucacagccuguUu................   57     1     seq
......................................................................uuugcaaaguaucacagccuguAu................   7      1     seq
.......................................................................uugcaaaguaucacagccu....................   1      0     seq
.......................................................................uugcaaaguaucacagccugu..................   5      0     seq
...........................................................................................ucugcgguauggcacaccg   1      0     seq
```
